# Population and conservation threats to the vulnerable Sarus crane *Grus antigone* in Nepal

**DOI:** 10.1002/ece3.10929

**Published:** 2024-02-07

**Authors:** Hari Prasad Sharma, Hem Bahadur Katuwal, Sandeep Regmi, Rajendra Narsingh Suwal, Rashmi Acharya, Amrit Nepali, Sabin KC, Bishnu Aryal, Krishna Tamang, Basudha Rawal, Amir Basnet, Bashu Dev Baral, Surya Devkota, Sagar Parajuli, Niraj Regmi, Pradip Kandel, Bishal Subedi, Hari Sharan Giri, Samjhana Kawan, Gokarna Jung Thapa, Bishnu Prasad Bhattarai

**Affiliations:** ^1^ Central Department of Zoology, Institute of Science and Technology Tribhuvan University Kathmandu Nepal; ^2^ Nepal Zoological Society Kathmandu Nepal; ^3^ Center for Integrative Conservation, Xishuangbanna Tropical Botanical Garden Chinese Academy of Sciences Mengla Yunnan China; ^4^ WWF Nepal Kathmandu Nepal; ^5^ Central Department of Environmental Science Tribhuvan University Kathmandu Nepal

**Keywords:** electrocution and collision, farmland, population, roosting site, transboundary conservation, wetland

## Abstract

Globally, biodiversity is declining due to habitat loss and degradation, over‐exploitation, climate change, invasive species, pollution, and infrastructure development. These threats affect the populations of large waterbird species, such as Sarus crane (*Grus antigone*), which inhabits agricultural–wetland ecosystems. Despite the burgeoning built‐up areas and diminishing agricultural and wetland spaces, scant research investigates the impact of these changing land uses on the globally vulnerable Sarus crane in Nepal. During the pre‐breeding season from April to June 2023, our comprehensive study meticulously scrutinized Sarus crane population status and factors associated with the occurrences and conservation challenges across 10 specific districts of Nepal. Our study documented a total of 690 individuals of Sarus cranes in five districts. The Lumbini Province has 685 individuals, occupying 11 roosting sites. Conversely, the remaining five districts have no Sarus cranes presence during this period. Wetland, farmland and built‐up areas exhibited a significantly positive influence on Sarus crane occurrences in the Lumbini Province. Additionally, we recorded 47 fatalities of Sarus cranes over the past 13 years in the Lumbini Province due to electrocution and collisions. Our study provides a baseline dataset crucial for developing conservation policies, particularly during the dry season when Sarus crane populations tend to congregate in larger flocks. The adaptation of the Sarus crane to urbanized landscapes exposes them to several anthropogenic threats in the coming days. Therefore, protecting wetlands and farmland areas and adopting transboundary conservation approaches are imperative for the long‐term conservation of the Sarus crane and its habitat.

## Graphical abstract

The first record of the population status of Sarus crane can be used as a baseline data for developing a management plan.

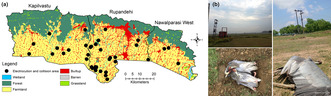

## 1 INTRODUCTION

Globally, the population of many bird species is declining (Ogada & Buij, 2011; Seress et al., 2012), and currently around 13% of total assessed bird species are facing extinction threats (BirdLife International, 2022). This decline is mainly due to various factors such as hunting, habitat loss, climate change, invasive alien species, some farmland practices, including crop intensification and agrochemical use, environmental pollution and poisoning, and infrastructure development (Clavero et al., 2009; Katuwal et al., 2023; Kociolek et al., 2011; Prakash et al., 2003; Seress et al., 2012; Spatz et al., 2023). Among these affected species, the Sarus crane (*Grus antigone*) in South Asia faces considerable challenges, especially in agricultural–wetland habitats, risking its survival (Inskipp et al., 2016; Katuwal et al., 2022; Sundar, 2011). However, there is limited knowledge regarding how this large‐sized waterbird utilizes the agricultural–wetland landscapes in South Asia.

Sarus crane is a resident crane species found in South Asian countries like Nepal, India, Pakistan, and Bangladesh (*antigone* subspecies, estimated population 8000–10,000), Southeast Asian countries such as Myanmar, Laos, Cambodia, Vietnam, and China (*sharpii* subspecies, estimated population 1300–1800), and Australia (*gilliae* subspecies, estimated population 10,000; Archibald et al., 2003; BirdLife International, 2022). Sarus crane primarily relies on paddy fields and wetlands for foraging and nesting (Borad et al., 2002; Gosai et al., 2016; Gulati & Rana, 2022; Katuwal, 2016; Mirande & Harris, 2019; Sundar, 2011; Tiwari et al., 2017). Its diet primarily includes invertebrates; small vertebrates such as fish, reptiles, amphibians, birds, and rodents; and plant materials such as roots, tubers, seeds, grains, and aquatic plants (BirdLife International, 2016). Despite its significance, the Sarus crane is classified as Vulnerable on the International Union for Conservation of Nature's Red List of Threatened Species assessments (BirdLife International, 2016).

In South Asia, India is home to the largest population of Sarus cranes (BirdLife International, 2022), but the overall population is rapidly declining (SoIB, 2023; Verma, 2019). However, the situation remains relatively unknown in Nepal. Although sporadic studies in Nepal have reported sightings in various regions, there has not been a comprehensive nationwide survey to determine their population status (Acharya, 2023; Aryal et al., 2009; Chhetri, 2023; DNPWC and GoN, 2021; Gosai et al., 2016; Katuwal, 2016; Suwal, 1999). Moreover, the regions in western Nepal where these cranes reside are threatened by encroachment and an anticipated decline in wetland and farmland areas, coupled with an increase in built‐up areas (Rimal et al., 2020). However, there is limited information available on how these land use variables affect the occurrences of Sarus crane.

Apart from habitat loss, factors like transmission and power lines contribute to increased mortalities of Sarus crane across India (Sundar & Choudhury, 2005), but detailed information about these incidents is lacking in Nepal, despite scattered newspaper reports and studies from the Greater Lumbini Area (Gosai et al., 2016; Katuwal, 2016; Poudel, 2022). The increasing tendency of the Sarus crane to forage and nest in human‐dominated landscapes poses multiple conservation challenges, including egg theft, hunting, canid predation, exposure to agrochemicals, and loss of essential roosting, nesting, and foraging sites (DNPWC and GoN, 2021; Gosai et al., 2016; Sundar, 2011; Suwal & Shrestha, 1992).

One significant challenge lies in the limited exploration and understanding of bird species outside protected areas, particularly in developing nations like Nepal (Inskipp et al., 2016; Katuwal, 2016; Katuwal et al., 2021). This highlights the urgent need for comprehensive studies and conservation efforts on Sarus crane, a nationally and globally threatened species, whose population status is unknown, and lacks systematic surveys in its habitats and threats across Nepal. Therefore, we aimed to identify the distribution, population, and conservation threats to the Sarus crane to fill the gaps in the Sarus crane study in Nepal. Our specific objectives were (1) to identify the population status of the Sarus crane in Nepal, (2) to map the major roosting sites of the species, (3) to investigate the factors affecting their occurrences, and (4) to assess fatalities due to electrocution and collisions. The findings from this field‐based research provide valuable insights for the scientific management of Sarus crane population when resources are limited, enabling the development of site‐specific conservation and management plans (Levenhagen et al., 2020).

## 2 MATERIALS AND METHODS

### 2.1 Study area

**FIGURE 1:**
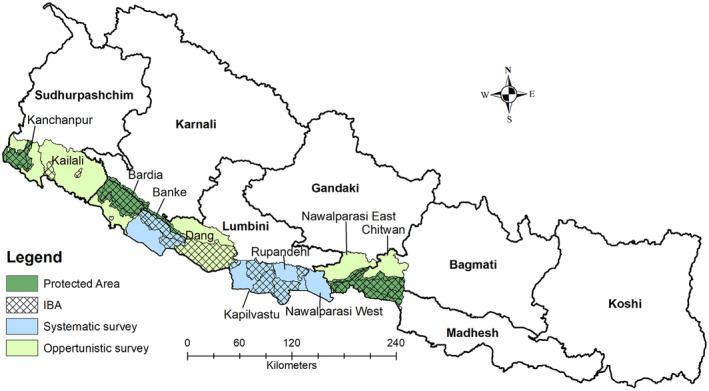
Sarus crane known distribution range and surveyed districts and provinces in Nepal. The four districts were systematically surveyed areas due to higher occurrences, whereas other six districts were opportunistically surveyed due to very less occurrences. See Section 2 for more details.

We conducted this study across the lowlands of Nepal from Chitwan to Kanchanpur districts, which are located within the Bagmati, Gandaki, Lumbini, and Sudhurpashchim provinces (Figure 1). The study area encompasses five Protected Areas and ten Important Bird and Biodiversity Areas (IBAs) and is part of the western Tarai Arc Landscape (DNPWC and BCN, 2022). Among them, Nawalparasi West, Rupandehi, and Kapilvastu districts are particularly categorized as the Greater Lumbini Area, which holds significant historical and religious importance as the birthplace of Lord Gautam Buddha in the Lumbini Province. The climate of the area is mixed tropical and sub‐tropical, with temperatures ranging from 10°C to 35°C. Additionally, the average monthly rainfall in the region varies from 75 to 150 mm.

Agriculture serves as the primary source of income in this region. During the monsoon season, rice is cultivated, while wheat, mustard, and a diverse range of vegetables are cultivated in winter. However, most people keep their land fallow in the summer season. Furthermore, the agricultural lands in this area are enriched with the planting of several tree species, such as *Bombax ceiba*, *Ficus bengalensis*, and *Eucalyptus* spp., which serve both local needs and commercial purposes. The study area is also home to various mammal species, including the golden jackal (*Canis aureus*), Bengal fox (*Vulpes bengalensis*), yellow‐throated marten (*Martes flavigula*), chital (*Axis axis*), barking deer (*Muntiacus vaginalis*), nilgai (*Boselaphus tragocamelus*), hog deer (*A. porcinus*), and leopard (*Panthera pardus*). Notable bird species found in the area include the Sarus crane, lesser adjutant (*Leptoptilos javanicus*), white‐rumped vulture (*Gyps bengalensis*), and reptile species include mugger crocodile (*Crocodylus palustris*) and golden monitor lizard (*Varanus bengalensis*).

### 2.2 Research design

We identified the potential distribution sites of Sarus crane within the Greater Lumbini Area and other relevant locations after consultation with local people and bird experts in the workshop organized on 26 April 2023. The study was systematically conducted in Lumbini Province as it has a stronghold of the Sarus crane population (Katuwal, 2016; Figure 1). We established a 5 km × 5 km grid, a standardized approach, and overlaid in the study area. However, we excluded the grids which constituted more than 70% forested areas particularly in the Chure region and other forest areas. This exclusion was decided based on the understanding that the Sarus cranes are not known to utilize forest habitats in Nepal (Grimmett et al., 2016). Finally, we surveyed 113 grids, including 20 grids in the Banke district and 93 grids within the Greater Lumbini Area. This comprehensive approach enabled the assessment of the Sarus crane population in each grid, including the identification of their roosting sites during the period of April to June 2023. Conversely, due to fewer recorded sightings, an opportunistic survey approach was adopted for the remaining six districts.

We applied a road count survey method using motorcycles by two observers to count the Sarus crane population accurately. Altogether 14 teams worked in the Greater Lumbini area, four in the Banke district, and two teams in the remaining districts of Lumbini Province. The observers traveled at a speed of 20–25 km per hour, covering all available roads within the designated grid. This method has been widely used and is effective in counting the population and measuring habitat variables of large waterbirds, including the Sarus crane (Koju et al., 2019; Sundar, 2011). Prior to the survey, we created a “kml” layer grids and their centroids and exported them to the MAPS.me application for all team members. It has facilitated precise location tracking during the survey. Careful attention was given to record the spatial locations of all Sarus crane sightings. Primarily, surveys were conducted in the morning from 6:00 am to 12 noon and in the evening from 2:00 pm to 6:15 pm. GPS devices and range finders were used to document accurately the precise presence points of Sarus cranes, which facilitated tracking of the exact location during the survey. Simultaneous surveys were conducted in adjacent grids to avoid potential instances of double counting, considering that Sarus cranes are large and easily observable in open areas. Additionally, the observers maintained communication via phone calls to synchronize their counts and shared information on various factors, including time, flock size, stable or flight directions, and other relevant details.

During the survey, we identified the roosting sites of Sarus cranes, which included rivers, wetlands, and agricultural lands. Besides that, we focused on determining the major roosting sites by closely examining the congregation and occurrences of Sarus cranes. We conducted surveys at the roosting sites during the morning, afternoon, and evening periods. While doing so, we implemented careful counting methods at each roosting site throughout the day and noted the highest count observed on that particular day. This approach ensured that we accurately captured the highest number of Sarus cranes present at the roosting sites.

### 2.3 Electrocution and collision incidents

We also investigated the electrocution and collision incidents during the survey. Furthermore, we interviewed the key informants and the local people regarding the electrocution and collision incidents to identify the number of fatalities and their locations.

### 2.4 Land use variables

To comprehend the factors influencing the Sarus crane occurrences, we downloaded the ESRI Sentinel‐2 land use land cover map having a 10 m resolution (Karra et al., 2021) and extracted the land use variables for each grid, including the wetland area (comprising water bodies), farmland, and built‐up areas of the studied area. These variables significantly influenced the occurrence of the waterbird in South Asian regions (Katuwal et al., 2022; Sundar et al., 2019). We extracted the area of these land use variables at 2.5 km buffer around the centroid of each grid. We measured the length of major roads and transmission lines (132 kV) in each grid.

### 2.5 Data analysis

We used direct count data from the grids including roosting sites and opportunistic sites conducted at almost the same time to assess the population of the Sarus crane in our study area. We exclusively utilized Sarus crane observations gathered from the grids within the three districts (Nawalparasi West, Rupandehi, and Kapilvastu) to understand the factors influencing occurrences, considering the constrained sightings in Banke. We used generalized linear modeling with a binomial distribution to identify the factors influencing the occurrences of Sarus crane. Our response variable was the presence–absence in each grid, and the predictors were the area of wetland (km^2^), built‐up areas (km^2^), farmland (km^2^), length of the transmission line (km), and major road (km) in each grid. Before conducting the analysis, we conducted a multi‐collinearity test and found the road to be highly correlated with built‐up areas (|r| > 0.7). Thus, we excluded length of the road from our analysis. All analyses were performed using the R Program (R Core Team, 2022). In addition, with the help of the key informants and the local people, we carefully mapped the electrocution and collision incidents for the last 12 years in the Lumbini Province (2010 to 2022).

## 3 RESULTS

**FIGURE 2:**
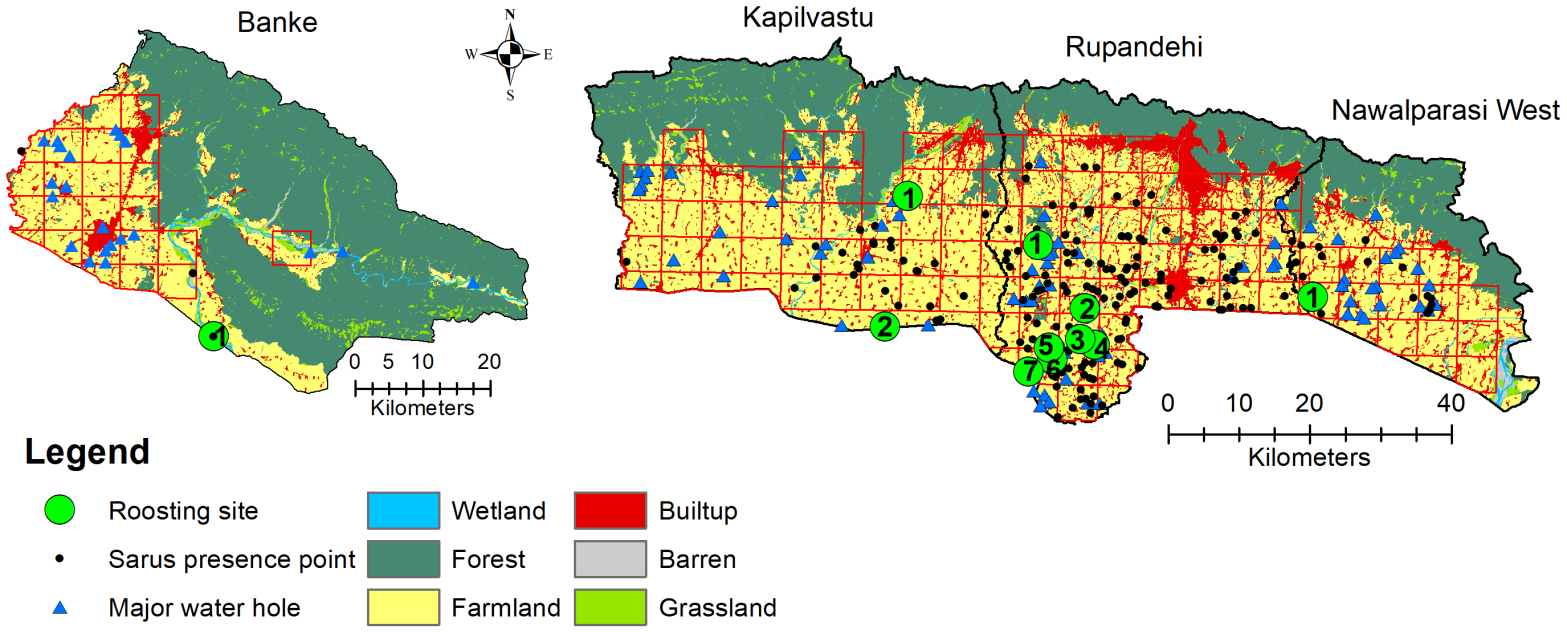
Pre‐breeding occurrences and major roosting sites of the Sarus crane observed in Lumbini Province. Opportunistic sighting in the Beldadi area of the Kanchanpur district is not shown here. 1–7 represents the major roosting sites in each district. Banke: (1) Bhagwanpur area; Kapilvastu district: (1) Jagdishpur Reservior, (2) Bajaha Taal; Rupandehi: (1) Farmlands along Bishnupura highway, (2) Khadiya, (3) Duimuhan area (confluence of Tinau and Dano river), (4) Majhgaun west, (5) Ardhauli East area, (6) Babai Durga Mandir area, and (7) Ajhmaghat area; Nawalparasi West: (1) Nandan Taal.

We observed the Sarus crane in all four systematically surveyed districts (Nawalparasi West, Rupandehi, Kapilvastu, Banke), but in only one of six opportunistically surveyed districts (Kanchanpur). We recorded the Sarus crane occurrence in 57 grids of lowlands from central to far‐western Nepal (Figure 2). Among these grids, the Sarus crane was recorded in 36 grids in Rupandehi, followed by Kapilvastu (12 grids), Nawalparasi West (six grids), and Banke (three grids) of Lumbini Province (Figure 2). Within these grids, flocks of Sarus crane ranged from one to 107 individuals.

### 3.1 Major roosting sites

We found a substantial number of waterholes as roosting sites for Sarus crane in the lowlands of Nepal (Figure 2). In the Banke district, we identified only one major roosting site in the Bhagwanpur area, two major roosting sites in Kapilvastu (Jagdispur Reservoir and Bajaha Taal), seven major roosting sites in Rupandehi (Bishnupura highway and Gaidahawa lake area, Khadiya ittabhatta area, Duimuhan (confluence of Tinau and Dano rivers), Majhgaun, Ardhauli East, Babai Durgamandir, and Ajhmaghat), whereas, in Nawalparasi West, we found a major roosting site at Nandan Lake (Figure 2). Most of these roosting sites lie along the Tianu and Dano rivers in the Rupandehi district. These areas are particularly important as they hold several presence locations of Sarus cranes. Some of these roosting sites are near the Nepal–India border (Figure 2).

### 3.2 Population status

**FIGURE 3:**
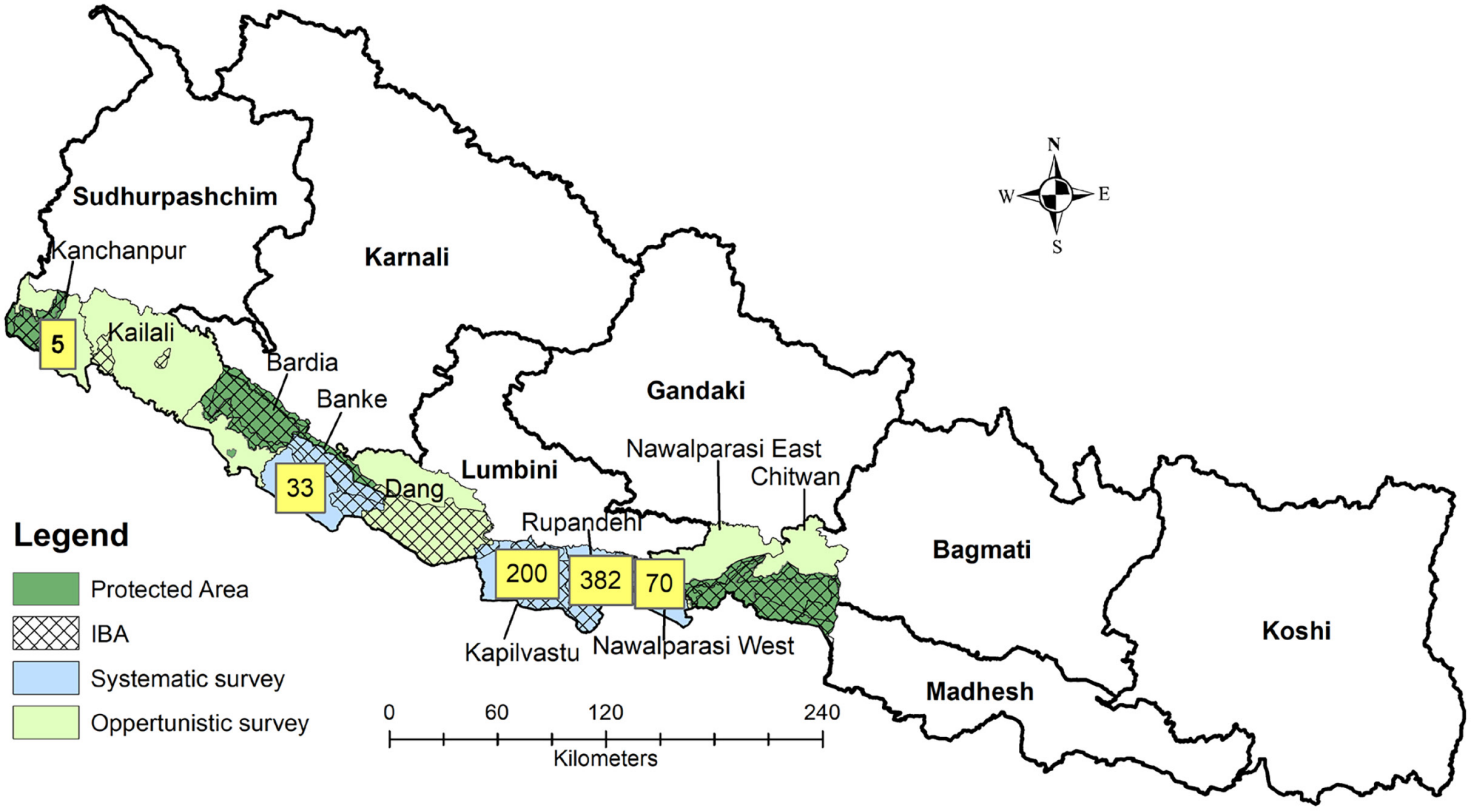
Sarus crane population during the pre‐breeding season of 2023 in Nepal.

Altogether, we recorded 690 individuals of Sarus crane in Nepal. This comprises 33 in Banke, 200 in Kapilvastu, 382 in Rupandehi, 70 in Nawalparasi West, and five in Kanchanpur district (Figure 3). However, there were no records of observations of target species in the following districts; Chitwan, Nawalparasi East, Dang, Bardia, and Kailali during this survey (Figure 3). Among the population, 7% (*n* = 49 individuals) were sub‐adults in the study area. We found the Sarus crane population was predominantly concentrated in and around lakes and river systems and few on farmland. We noticed some individuals close to the Nepal–India border in Rupandehi (Ajhmaghat), Kapilvastu (Bajaha Taal), and Banke (Bhagwanpur area) of Nepal.

### 3.3 Factors affecting the occurrences of Sarus crane

The average area of farmland across the grids was 15.001 ± 3.165 (SD) km^2^ (range = 3.785–18.478), wetland area was 0.191 ± 0.247 (SD) km^2^ (range = 0–0.998), and the average area of the built‐up area was 2.783 ± 1.918 (SD) km^2^ (range = 0.822–12.374). The average length of the transmission line across the study grids was 1.776 ± 2.316 km.

The wetland, farmland, and built‐up area significantly influenced the occurrences of Sarus crane (Table 1). Although not statistically significant, Sarus crane occurrences exhibited a negative association with the length of the transmission line (Table 1)

**TABLE 1:** The factors influencing the occurrences of Sarus crane in the Lumbini Province using a generalized linear model with a binomial distribution.

Parameters	Estimate	SE	*z*	*p*
Intercept	0.234	0.277	0.846	.398
Wetland area	1.083	0.402	2.7	<.001
Built‐up area	1.242	0.416	2.984	.002
Farmland area	2.01	0.575	3.493	<.001
Transmission line length	−0.483	0.262	−1.841	.06

*Note*: Variables used in the model were Sarus crane presence/absence across the grid as the response variable, and area of wetland area (km^2^), built‐up area (km^2^), farmland (km^2^), and length of the transmission line (km) as predictive variables for model construction.

### 3.4 Electrocution and collision threats

**FIGURE 4:**
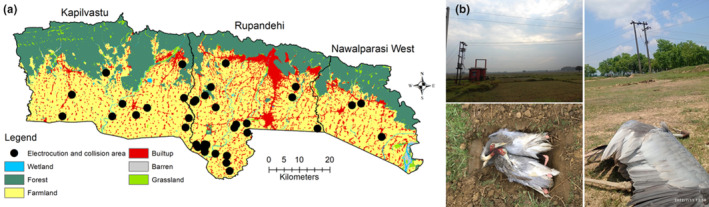
Fatalities of Sarus crane due to electrocution and collisions (2010–2023) in the Greater Lumbini Area, Lumbini Province. The figure displays the approximate locations (a), transmission lines crossing the farmland, and the number of individuals killed during this period (b). Photographs by Kailash Jaiswal and Arjun Kurmi.

We documented a total of 47 fatalities of Sarus crane from 2010 to 2023 due to electrocution and collision incidents within the Greater Lumbini Area only (Figure 4a). All of these incidents occurred within the farmland habitat of the Sarus crane (Figure 4b).

## 4 DISCUSSION

We confirmed the first nation‐wide population status of the Sarus crane during the pre‐breeding season in the lowlands of Nepal. Our comprehensive survey not only pinpointed key roosting sites but also meticulously assessed the distribution of these birds across different districts. Through our investigation, we unveiled critical factors influencing their presence, highlighting electrocution and collisions as primary threats to their conservation. One of the important findings of our study is the identification of Lumbini Province as the primary major hotspot for the Sarus crane in Nepal.

The lowland areas where Sarus crane is found fall under the global distribution range of Sarus crane (BirdLife International, 2016). Not only in this study, the Sarus crane in Nepal has been recorded from central to far‐western regions (Aryal et al., 2009; Gosai et al., 2016; Katuwal, 2016; Tiwari et al., 2017). The study identified Rupandehi and Kapilvastu districts as significant hotspots for the Sarus crane, indicating a concentrated presence of the species. The higher concentration in these areas might be due to the presence of the larger farmlands and the wetlands in close proximity that provides abundant resources for the species. Nawalparasi West was also identified as a closely monitored hotspot. In comparison to previous reports by other researchers (Gosai et al., 2016; S. Gnawali (Personal communication, May 2023)), our study documented a wider distribution and a higher number of Sarus crane population in the Nawalparasi district. This variation might be due to variations in observation time, area coverage, and efforts. The presence of a small population of Sarus crane in the Banke district during this time was lower than previously reported (Tiwari et al., 2017), which could be attributed to differences in observation methods and study periods. Multiple counting of Sarus cranes is also a possibility in previous studies, as the number of individuals was counted at different times.

Prior to our study, there has been no systematic assessment of the Sarus crane population in Nepal. Previous studies focused on smaller areas of Rupandehi, Kapilvastu, Nawalparasi, and Banke districts. Inskipp et al. (2016) assumed a population size ranging from 450 to 700+ individuals, while Karmacharya et al. (2020) predicted approximately 1500 individuals in the Lumbini area using GIS modeling. In contrast, our comprehensive study covering the entirety of Nepal confirmed a population count of 690 individuals during the pre‐breeding season. We consider this count more accurate due to our methodology of counting individuals at roosting sites and grids simultaneously, with multiple teams and specific time constraints, enabling a more precise estimate. Notably, previous studies had limited personnel and seldom surveyed all the roosting sites simultaneously, making our population count the highest ever recorded for each district in Nepal, establishing a valuable baseline for future reference. However, it is essential to acknowledge that the population might be higher than reported. Over 100 individuals were counted in Bajaha Lake of the Kapilvastu district during the last winter of 2022–2023 (Samjhana Kawan personal observation), while we recorded very few during our visit, possibly due to migration to the Indian side due to drying of wetlands in Nepal. Additionally, large flocks observed in the Ajhmaghat area of the Dano River in Rupandehi, near the India border, suggest local migration, emphasizing the importance of transboundary research and conservation efforts. Although the overall population of the Sarus crane is declining in India (SoIB, 2023), it is worth noting that the Sarus crane population is increasing in some regions of north India (Verma, 2019), yet a long‐term systematic monitoring is needed to understand their population trend in Nepal.

The Sarus crane is typically associated with wetland habitats but is also frequently found in agricultural landscapes (Katuwal, 2016; Sundar, 2011). Wetland and farmland areas were identified as key factors influencing their occurrence during the pre‐breeding season, with habitat utilization varying across seasons. Wetland and farmland areas play a crucial role as foraging and breeding grounds for the Sarus crane (Gosai et al., 2016; Katuwal, 2016; Sundar, 2011). However, these vital habitats for the Sarus crane are gradually diminishing in the Lumbini Province (Chhetri, 2023). The drying of wetlands has forced the cranes to migrate further south, often crossing border areas where water sources are available. To ensure the conservation of the species, it is imperative to prioritize the maintenance and protection of wetlands during the summer season to provide sufficient water access during this critical period. Additionally, we also observed a positive influence of built‐up areas. It should be noted that this landscape is anthropogenized, with settlements and small‐scale industries regularly found in farmland (Katuwal et al., 2022; Rimal et al., 2020), forcing the Sarus crane to adapt to these humanized environments. However, such adaptation introduces several anthropogenic challenges, including hunting and egg and chick vandalism. If the expansion of these built‐up areas continues, it could have a detrimental effect on the presence of the Sarus crane in the future. Therefore, a comprehensive study should be undertaken to determine the threshold that the Sarus crane can tolerate in urbanized landscapes.

Electrocution and collisions pose significant threats to Sarus cranes in the Lumbini province. The presence of transmission lines passing through their important roosting and breeding habitats in agricultural lands and wetlands increases the likelihood of these incidents. We reported several electrocution and collision incidents and the negative influences of the lengths of the transmission lines on the occurrences of the Sarus crane (Chhetri, 2023). Studies show that about 1% of Sarus cranes in Uttar Pradesh, India, die annually due to electrocution (Sundar & Choudhury, 2005). Additionally, electrocution and collisions have caused approximately 40% of whooping crane deaths and around 2% of common crane deaths in other regions (Brown et al., 1987; Ferrer et al., 1991). Similar incidents have been observed in Nepal across various bird species (Hamal et al., 2023). Conservation efforts in the Greater Lumbini Area should prioritize addressing these anthropogenic threats, including wetland habitat loss, and the risk of electrocution and collisions.

## 5 CONCLUSION AND CONSERVATION IMPLICATIONS

Our study provides valuable insights into the population and roosting sites of the Sarus crane during the pre‐breeding season in Nepal, with Lumbini Province identified as a significant hotspot for the species. The species favor wetlands over agricultural areas during the dry season, and we also found the positive influence of these variables, including the built‐up areas. However, increasing the urbanized landscape, particularly within farmland and wetland areas, may expose several anthropogenic threats to the Sarus Crane in near future. In addition, we showed that the electricity wire networks as major conservation threats to the Sarus crane. These findings have crucial implications for guiding conservation strategies to ensure the long‐term survival of the Sarus crane in Nepal. Conservation efforts should prioritize the protection of roosting sites throughout the Greater Lumbini Area, conduct regular population assessments during the breeding season, and implement seasonal management of wetland habitats, promoting wetland establishment along agricultural landscapes. To safeguard the species, transmission line planning should avoid crucial farmland and Sarus crane hotspots. Encouraging agricultural–wetland policies that discourage haphazard urbanization of agricultural lands will also be beneficial. Given the proximity of some roosting sites to the Nepal–India border, transboundary research and conservation initiatives are essential. Additionally, the utilization of satellite tagging can provide valuable information on the species movement ecology. Implementing these measures will effectively protect the Sarus crane population and its critical agricultural–wetland habitats in Nepal.

## AUTHOR CONTRIBUTIONS

**Hari Prasad Sharma:** Conceptualization (equal); data curation (equal); formal analysis (equal); funding acquisition (equal); investigation (equal); methodology (equal); project administration (equal); validation (equal); writing – original draft (equal); writing – review and editing (equal). **Hem Bahadur Katuwal:** Conceptualization (equal); data curation (equal); formal analysis (equal); methodology (equal); writing – original draft (equal); writing – review and editing (equal). **Sandeep Regmi:** Conceptualization (equal); data curation (equal); formal analysis (equal); writing – original draft (equal); writing – review and editing (equal). **Rajendra Narsingh Suwal:** Conceptualization (equal); writing – review and editing (equal).**Rashmi Acharya:** Investigation (equal); methodology (equal); writing – review and editing (equal). **Amrit Nepali:** Investigation (equal); writing – review and editing (equal). **Sabin KC:** Investigation (equal); writing – review and editing (equal). **Bishnu Aryal:** Investigation (equal); writing – review and editing (equal). **Krishna Tamang:** Investigation (equal); writing – review and editing (equal). **Basudha Rawal:** Investigation (equal); writing – review and editing (equal). **Amir Basnet:** Investigation (equal); writing – review and editing (equal). **Bashu Dev Baral:** Investigation (equal); writing – review and editing (equal). **Surya Devkota:** Investigation (equal); writing – review and editing (equal). **Sagar Parajuli:** Investigation (equal); writing – review and editing (equal). **Niraj Regmi:** Investigation (equal); writing – review and editing (equal). **Pradip Kandel:** Investigation (equal); writing – review and editing (equal). **Bishal Subedi:** Investigation (equal); writing – review and editing (equal). **Hari Sharan Giri:** Investigation (equal); writing – review and editing (equal). **Samjhana Kawan:** Investigation (equal); writing – review and editing (equal). **Gokarna Jung Thapa:** Writing – review and editing (equal). **Bishnu Prasad Bhattarai:** Conceptualization (equal); investigation (equal); methodology (equal); writing – review and editing (equal).

## CONFLICT OF INTEREST STATEMENT

Authors declare no conflict of Interest.

## Data Availability

The data used for this research will be available at Dryad with DOI link for readers after the manuscript is accepted. For editors and reviewers, it is available at Dryad's link https://datadryad.org/stash/share/WSroCFf5tZMMmS3Xp‐NqSRgRtXjSeUsPc4NfFw1wtoA.
